# Ischaemic Preconditioning and Intermittent Clamping Does not Influence Mediators of Liver Regeneration in a Human Liver Sinusoidal Endothelial Cell Model of Ischaemia-Reperfusion Injury

**DOI:** 10.4021/gr449w

**Published:** 2012-05-20

**Authors:** Dhanwant Gomez, J.. Lance Burn, Ann Graham, Shervanthi Homer-Vanniasinkam, K.. Rajendra Prasad

**Affiliations:** a Department of Hepatobiliary Surgery and Transplantation, St. James’s University Hospital, Leeds, UK; b Section of Oncology, University of Sheffield, UK; c Department of Biomedical Sciences, University of Bradford, Bradford, UK; d Department of Vascular Surgery, Leeds General Infirmary, Leeds, UK

**Keywords:** Ischaemic reperfusion, Ischaemic preconditioning, Regeneration, Liver

## Abstract

**Background:**

The role of surgical technique on liver regeneration following surgery remains inconclusive. The aim of the study was to assess the effect of ischaemic preconditioning (IPC) and intermittent clamping (IC) on mediators of regeneration produced by human liver sinusoidal endothelial cells (SECs), using an in vitro hypoxia-reoxygenation model to mimic ischaemia-reperfusion injury (IRI).

**Methods:**

Following extraction from samples obtained from liver resection (n = 5), confluent culture flasks of SECs were subjected to IRI (1 hour hypoxia + 1 hour reoxygenation), IPC prior to IRI (10 minutes hypoxia + 10 minutes reoxygenation + 1 hour hypoxia + 1 hour reoxygenation), IC (15 minutes hypoxia + 5 minutes reoxygenation x 3 + 1 hour reoxygenation) and compared to controls. The production of various mediators was determined over 48 hours.

**Results:**

Interleukin (IL)-6, IL-8, granulocyte-colony stimulating factor (G-CSF) and hepatocyte growth factor (HGF) were produced by SECs. Both IPC and IC did not significantly influence the profile of IL-6, IL-8, G-CSF and HGF by SECs compared to IRI over the study period.

**Conclusion:**

IPC and IC did not influence the production of pro-regenerative mediators in a SECs model of IRI. The role of surgical technique on liver regeneration remains to be determined.

## Introduction

Since the establishment of hepatic resection as the optimal treatment choice for both primary and secondary malignancies, investigators have focused their research on alleviating the inevitable phenomenon of ischaemia-reperfusion injury (IRI) following liver surgery and promoting regeneration of the remnant liver. Besides causing parenchymal cell injury and liver dysfunction that leads to morbidity and mortality post-surgery, IRI can significantly impair liver regeneration [1].

Liver regeneration is a complex and multi-factorial process that is mediated by interactions between regenerative cytokines, growth factors and metabolic demand of the liver following surgery and IRI. Briefly, following hepatectomy, the regeneration process is initiated by the regenerative cytokine network which includes tumour necrosis factor-alpha (TNF-α) and interleukin (IL)-6 [2, 3]. Following stimulation from cytokine triggering mechanisms, TNF-α binds to its receptor on non-parenchymal liver cells (Kupffer cells and sinusoidal endothelial cells (SECs)) and stimulates the production of IL-6, [4]via the activation of the transcription factor nuclear factor-kappa B (NF-κB) [5]. IL-6 acts directly on hepatocytes by binding to the IL-6 receptor complex and induces the translocation of signal transducer and activator of transcription-3 (STAT-3) to the nucleus. Following this, the two main growth-promoting signalling systems; hepatocyte growth factor (HGF) and its receptor (Met) and the epidermal growth factor (EGF) receptor and its large family of ligands which includes transforming growth factor-alpha (TGF-α) and EGF, [6] initiates a cascade of events that leads to progression of the cell cycle, culminating in the synthesis of DNA and subsequent cellular mitosis.

Various investigators have researched the role of surgical techniques to limit the detrimental effects of IRI and improve liver regeneration. These techniques include ischaemic preconditioning (IPC) and intermittent clamping (IC) of the portal triad, which are used in clinical practice to limit blood loss during liver resection. IPC involves a short period of vascular occlusion of both the arterial and portal venous inflow to the liver followed by reperfusion prior to a more prolonged inflow occlusion during parenchymal transection, while IC consists of repeated cycles of 15 to 30 minutes of ischaemia followed by 5 to 10 minutes of reperfusion during the resection phase.

The hypothesis of this study is that surgical techniques (IPC and/or IC) up-regulates the production of cytokines and growth factors by human liver SECs involved in liver regeneration. Following extraction, SECs were exposed to various hypoxia and reoxygenation settings to mimic ischaemia and reperfusion, and the effects of IPC and IC on the production of cytokines and growth factors were determined.

## Materials and Methods

### SECs extraction and culture

Human SECs and hepatocytes were extracted from liver specimens obtained from the Hepato-biliary and Transplantation Unit, St. James’s University Hospital, Leeds. Ethical approval was obtained from the Leeds (East) Regional Ethics Committee (reference: 03/316), with informed consent obtained from all patients involved.

Hepatocytes were isolated using a two step perfusion and digestion method described by Seglen [7]. After obtaining the liver specimen, 1 litre of sterile phosphate-buffered saline (PBS) at 4 °C was syringed through visible blood vessels on the cut surfaces to flush out the patient’s blood and its constituents. The specimen was then transported in University of Wisconsin transplantation fluid on ice (4 - 8 °C ) to the laboratory. All procedures were performed in a laminar-flow hood to minimise the risk of contamination. The liver specimen was placed on a warm stage and held in place with pipette tips. The visible veins were cannulated with the perfusion tubing. The liver specimen was perfused with liver perfusion medium (Invitrogen, Paisley, UK) through several cannulae at 60 - 100 mL/min at 37 °C for 30 minutes. The tissue was then perfused with liver digest media (Invitrogen, Paisley, UK) at 60 - 100 mL/min at 37 °C for 30 minutes. The digested liver was then cooled by immersion in suspension buffer (Dulbeccos minimum essential medium and 2% bovine serum albumin). The liver specimen was then cut open and cells released by gentle scraping with a scalpel. The undigested material and any large fragments are removed by filtration through a coarse sieve (pore size 1mm) into a freezer-cooled beaker, followed by a cell dissociation sieve (pore size 250 µm). The filtrate was collected in a freezer-cooled beaker and divided into freezer-cooled 50 mL tubes for centrifugation. The cell suspension was centrifuged at 50 g, at 4 °C , for 2 minutes, to pellet the dense viable hepatocytes. The supernatant was then collected, toped up to 50 mL and centrifuged at 50 g, at 4 °C, for 2 minutes. Following this, the supernatant was collected, toped up to 50 mL and centrifuged at 800 g, at 4 °C, for 8 minutes. The pellet was re-suspended in 30 mL of Solution A (Hanks Balanced Salt Solution, 5%FCS, 20 mM HEPES, gentamicin and amphotericin). Then, 15 mL of re-suspended cells was added to 50 mL tubes with 15 mL 34% Iodixanol in Solution A. This was mixed to obtain a 17% Iodixanol suspension solution with a relative density (r) of 1.095 g/mL, 15 mL of 8.5% Iodixanol was carefully added on the top, followed by 3 mL of Solution A to the top. The cell suspension was centrifuged at 3000 g, at 4 °C , for 20 minutes. The middle band, which contains both Kupffer cells and SECs were recovered. The cells were re-suspended in Solution A and centrifuged at 1000 g, at 4 °C , for 3 minutes. The supernatant was discarded and the cell pellet washed again in Solution A. The cell pellet was re-suspended in 1.5 mL of Solution A (4 °C ) in an eppendorf, 200 µl of anti-CD4 (clone RPA-T4, P.A.R.I.S., France) Dynabeadä (Invitrogen, Paisley, UK) suspension (2 x 10^8^ beads/mL in solution A) was added to the cell suspension. The dynabead/cell suspension was mixed with end over end rotation at 4 °C for 20 minutes. This suspension was then diluted to 10 mL in Solution A in a test tube and placed in a magnetic particle concentrator (MPC) for 3 minutes at room temperature. During this time period the dynabeads, now attached to CD4-expressing cells came to rest on the side of the universal container adjacent to the MPC. Following this, the supernatant was removed and the CD4-expressing cells remain in the test tube, 10 mL of Solution A was added to the universal container, and the procedure was repeated three times. The remaining rosetted cells were then re-suspended in supplemented endothelial basal cell media (EBM-2, (Clonetics, Wokingham, UK)), and CD4-expressing SECs were cultured on collagen IV coated 75 cm^2^ culture flasks.

SECs were passaged when a confluent monolayer was observed, at a ratio of 1:3. Following removal of media, cells in a 75 cm^2^ tissue culture flask were washed twice with PBS, and then trypsin/ethylenediamine-tetraacetic acid (EDTA) solution was added. When the cells were observed microscopically to be losing cell-cell contact (“rounding up”), the trypsin/EDTA solution was removed. The tissue flask was then incubated at 37 °C for 1 minute. Following this, the flask was tapped against the palm of the hand to detach the cells from the base of the flask, and once this had been observed microscopically, the cells were re-suspended in the desired volume of EBM-2 media and divided equally in three 75 cm^2^ culture flasks. The media was routinely changed after 24 hours, and again every 48 hours until cells became confluent for further passage.

SECs were grown to confluence in 75 cm^2^ culture flasks. Experiments were performed on four confluent flasks up to passage 3 that contained around 24.5 x 10^6^ SECs.

### SECs characterisation

Homogenous populations of SECs were confirmed on microscopy by the presence of a monolayer of morphologically distinct endothelial cells with “cobblestone” morphology. In addition, SECs were characterised by immunohistochemistry and the expression of von Willebrand factor (vWF) and CD31, also known as platelet endothelial cell adhesion molecule (PECAM)-1. SECs in culture expressed both vWF [8] and CD31 [9], and their function was determined by metabolic labelling, based on the uptake of fluorescein isothiocyanate-labeled formaldehyde-treated serum albumin (FITC-FSA) [10].

### Detection of CD31 expression

SECs were grown on 13 mm diameter glass cover-slips and washed twice with PBS prior to fixing. Cells were fixed for staining with 4% formaldehyde. Fixed cells were washed on three occasions with PBS, 5 minutes per wash with occasional agitation. The non-specific antigens were blocked with PBS containing 10% goat serum and 10% casein for 10 minutes. The primary (1^o^) mouse anti-human PECAM (clone 9G11, R and D systems, UK) antibody solution was prepared at the dilution of 1:50 in 2% goat serum in PBS. Cells were incubated with the primary antibody solution for 1 hour at room temperature in a humidified atmosphere. The cells are then washed on three occasions with PBS, 5 minutes per wash with occasional agitation. Following this, the secondary (2^o^) FITC conjugated goat anti-mouse antibody (Dako) solution was prepared at the dilution of 1:100 in 2% horse serum in PBS. The cells were incubated with the secondary antibody solution for 1 hour at room temperature in a humidified atmosphere. The cells were rinsed in three changes of PBS, 5 minutes per wash with occasional agitation. Cover slips were mounted on to slides, cells down, using 1:1 glycerol to PBS mounting fluid. Staining was observed using a microscope equipped with fluorescence. All photographs were taken using a digital camera (JVC, KY-F1030).

### Detection of vWF expression

SECs were grown on 13 mm diameter glass cover-slips and washed twice with PBS prior to fixing. Cells were fixed for staining with 4% formaldehyde. The cell membranes were permeabilised with absolute methanol at -20 °Cfor 10 minutes. Fixed cells were washed on three occasions with PBS, 5 minutes per wash with occasional agitation. The non-specific antigens were blocked with PBS containing 10% goat serum and 10% casein for 10 minutes. The primary (1^o^) rabbit anti-human vWF (Dako) antibody solution was prepared at the dilution of 1:50 in 2% goat serum in PBS. Cells were incubated with the primary antibody solution for 1 hour at room temperature in a humidified atmosphere. The cells are then washed on three occasions with PBS, 5 minutes per wash with occasional agitation. Following this, the secondary (2^o^) swine anti-rabbit antibody solution was prepared at the dilution of 1:100 in 2% goat serum in PBS. Cells were incubated with the secondary antibody solution for 1 hour at room temperature in a humidified atmosphere. The cells were rinsed in three changes of PBS, 5 minutes per wash with occasional agitation. Cover slips were mounted on to slides, cells down, using 1:1 glycerol to PBS mounting fluid. Staining was observed using a microscope equipped with fluorescence.

### Metabolic labelling - uptake of FITC-FSA

The ability to take up FITC-FSA by scavenger receptors has been used as a marker for SECs in-vitro [10]. Prior to SECs incubation with FITC-FSA, three solutions were made: Buffer 1 (0.2 M Sodium Carbonate Buffer pH 10); Buffer 2 (0.2 M Sodium Carbonate Buffer pH 9.5); and Nairn’s solution (34 g of sodium chloride, 1.38 g of NaH_2_PO_4_.H_2_O, 4.28 g of anhydrous Na_2_HPO_4_ and 4l of distilled water (adjusted to a pH of 7.4)). FSA was prepared by mixing 9 mL of Buffer 1 with 1 mL of 37% formaldehyde and 1g of BSA and incubated at 4 °C for 3 days. This solution was then dialysed (12,000 MW) against 4 litres of 0.1 M sodium chloride for two days with two changes of sodium chloride. This produced a 10% FSA solution, 10% FSA was added to Buffer 2 and made up to 20 mL. Following this, 200 mg of FITC was added and mixed well. This solution was stored overnight at 4 °C. After overnight storage, this solution was centrifuge at 10,000 rpm for 10 minutes and then dialysed against 4 litres of Nairn’s solution for 3 days at 4 °C with 3 changes of Nairn’s solution. The FITC-FSA was now ready.

SECs were grown on 13 mm diameter glass cover-slips. The cultured cells were washed twice with PBS, 100 mg/mL of FITC-FSA in supplemented EBM-2 media was required and this was equivalent to the addition of 2 ml of 50 mg/mL FITC-FSA in 10 mL of supplemented EBM-2 media. The cells were incubated with FITC-FSA for 2 hours at 37 °C in the incubator. The cells are then washed on three occasions with PBS, 5 minutes per wash with occasional agitation. The cells were fixed with 4% formal buffered saline and stored at 4 °C until ready to be viewed. Cover slips were mounted on to slides, cells down, using 1:1 glycerol to PBS mounting fluid. The uptake of FITC-FSA was observed using a microscope equipped with fluorescence, FITC was activated using blue light (450 - 490 nm).

### Experimental design

#### IRI

**Figure 1:**
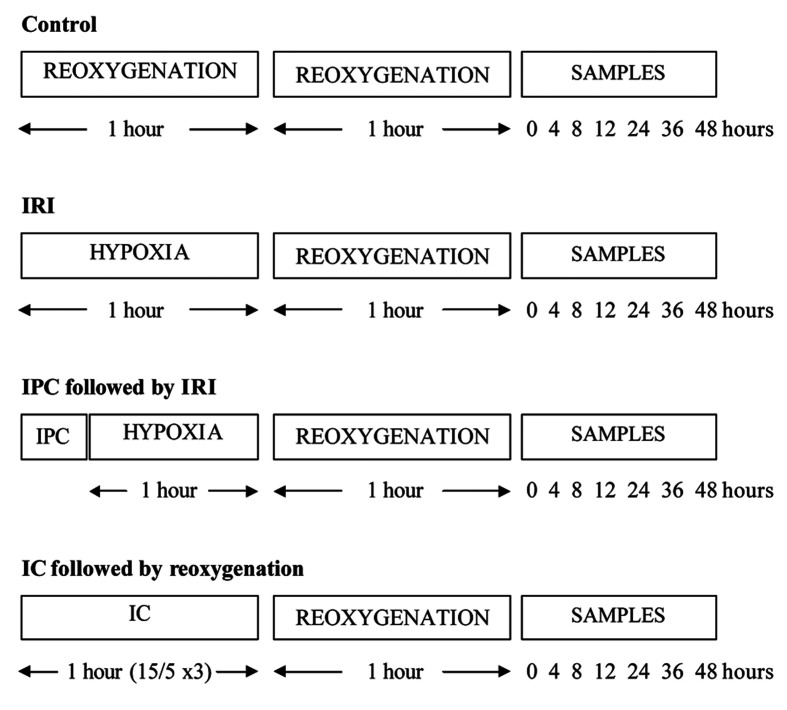
Ischaemia and reperfusion protocols used in this study.

The ischaemia and reperfusion periods were determined at 1 hour of hypoxia and 1 hour of re-oxygenation, respectively (Fig. 1). All hypoxia and reoxygenation experiments involved the use of a microaerophilic chamber with O_2_ concentration routinely measured and found to be < 0.1%. This chamber contained inlet gases of nitrogen and carbon dioxide at 37 °C. An appropriate amount of supplemented EBM-2 media were placed inside the chamber for 24 hours prior to the experiments, to allow oxygen to diffuse out of the media. Following this, flasks that required hypoxia treatment had their oxygenated media removed, and were incubated in the microaerophilic chamber for 1 hour in hypoxic media. The control flasks experienced an identical number of media changes, but otherwise remained in the 5% CO_2_ incubator at 37 °C for identical time periods. At the end of the hypoxic period, flasks were removed from the chamber, and the media discarded. The media was also discarded from the control. Following this, samples had EBM-2 media added for the reoxygenation period, and were returned to the 5% CO_2_ incubator. Following the 1 hour reoxygenation period, 100 µmL of media were collected at relevant time points and stored in eppendorfs at -80 °Cuntil processed.

#### IPC and IC

The IPC protocol was determined at 10 minutes of hypoxia followed by 10 minutes of re-oxygenation. Culture flasks for IPC had their media removed, and were incubated in a microaerophilic chamber for 10 minutes in hypoxic EBM-2 media. After hypoxic preconditioning, these samples were removed from the chamber, and the hypoxic media was replaced with EBM-2 media. They were placed back in the 5% CO_2_ incubator at 37 °C for the reoxygenation preconditioning period for 10 minutes. The samples were then exposed to 1 hour of hypoxia and 1 hour of reoxygenation.

The IC protocol was set at 15 minutes of hypoxia followed by 5 minutes of reoxygenation, and this cycle was performed a total of 3 times, to equal 1 hour. During the hypoxia phase of IC, culture flasks were incubated in a microaerophilic chamber with O_2_ concentration < 0.1% for 15 minutes in hypoxic EBM-2 media. After the hypoxic phase, these flasks were removed from the chamber, and the hypoxic media was replaced with EBM-2 media. They were placed back in the 5% CO_2_ incubator at 37 °C for 5 minutes for the reoxygenation period. This cycle was then repeated two further cycles. The samples were then exposed to 1 hour of reoxygenation.

### Multiplex cytokine and growth factor analysis

Growth factors and cytokines involved in the liver regeneration cascade that were assessed in this study were IL-1β, IL-1 receptor antagonist (IL-1ra), IL-6, IL-8, EGF, TGF-α, granulocyte-colony stimulating factor (G-CSF), TNF-α and HGF. Cytokine analysis kits (Human Cytokine LINCO*plex* kit (Linco, cat no. hCYTO-60K) and Human Adipocyte LINCO*plex* kit (Linco, cat no. HADCYT-61K)) were used. Assays were run in duplicates according to the manufacturers’ protocol. Data was collected using the Luminex ® 100 ™ IS System (Luminex, cat no. CN-L003-01).

### Statistical analysis

All concentration values are presented as the mean (standard error of the mean). Comparison between groups at each time-point over the 48 hour period was assessed using the Mann Whitney U Test. Statistical analyses were performed using the SPSS for Windows™ version 15.0 (SPSS Inc, Chicago, Ill, USA), and statistical significance was taken at the 5% level.

## Results

### SECs

**Figure 2:**
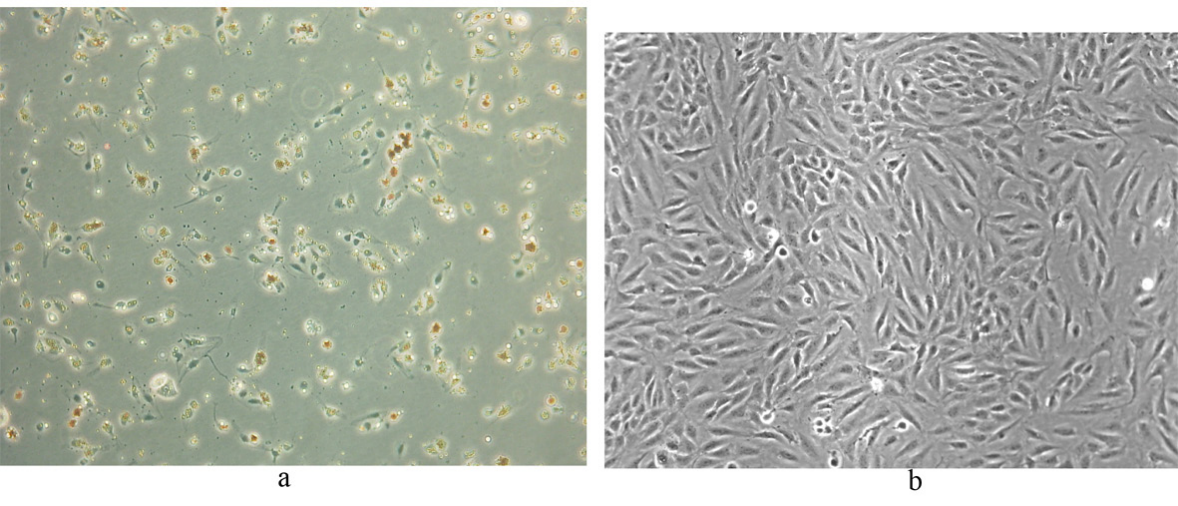
Human liver SECs. (a) Appearance of SECs at day 1 of extraction (phase contrast x 100).

**Figure 3:**
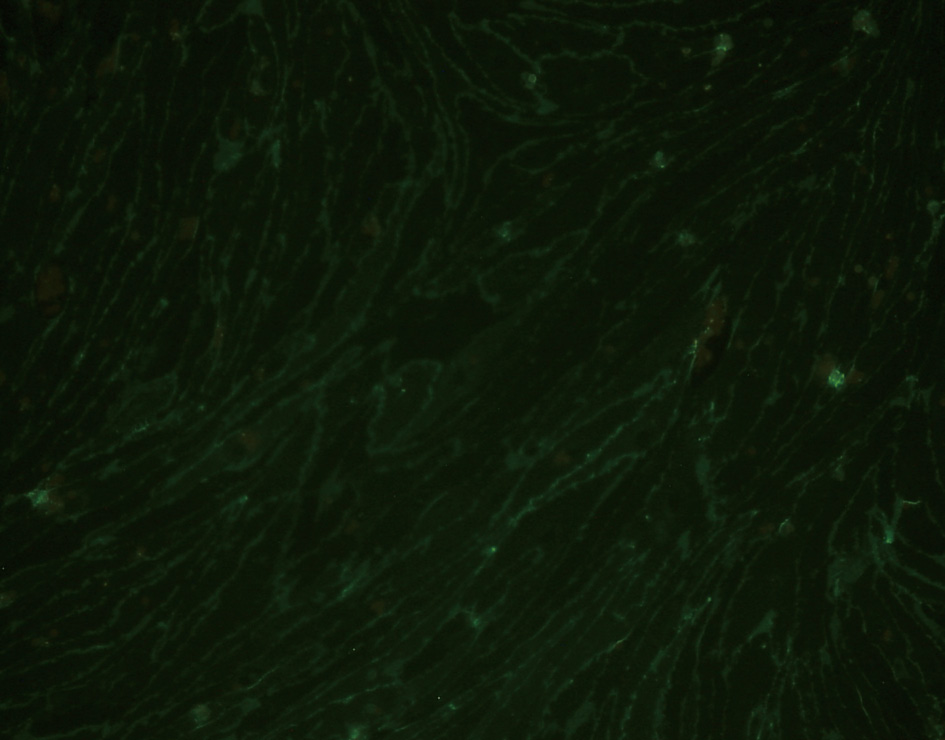
Immunohistochemistry demonstrating the expression of CD31 in human liver SECs (fluorescence x 200).

**Figure 4:**
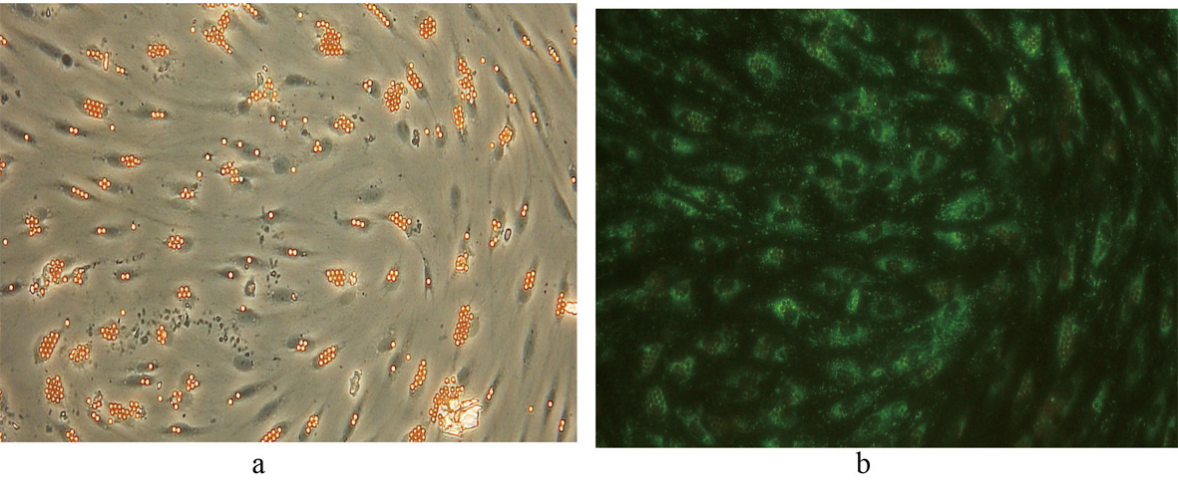
Immunohistochemistry demonstrating the expression of vWF in SECs. Phase contrast image of the same field of view is shown for comparison. (a) Phase contrast x 200. (b) Fluorescence x 200.

**Figure 5:**
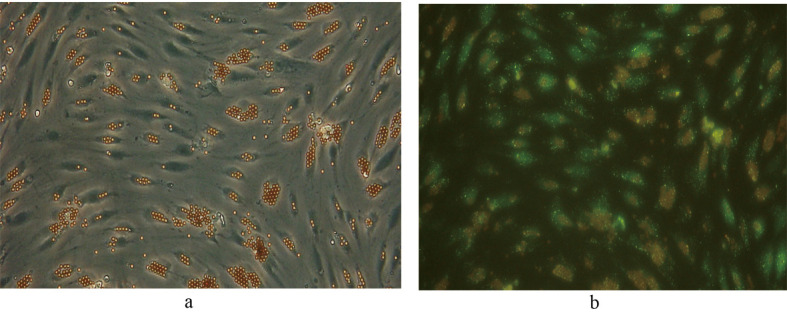
Fluorescent image demonstrating the uptake of FITC-FSA in human liver SECs. Phase contrast image of the same field of view is shown for comparison. (a) Phase contrast x 200. (b) Fluorescence x 200.

Following selectively isolating CD4 positive SECs, confluent culture flasks of SECs demonstrated “cobblestone” morphology on microscopy (Fig. 2 a, b). After all extractions (n = 5), SECs were determined by the expression of CD31 (Fig. 3) and vWF (Fig. 4), and the uptake of FITC-FSA (Fig. 5) using immunohistochemistry.

### Effects of IRI, IPC and IC

Of the mediators measured, IL-6, IL-8, G-CSF and HGF were produced by SECs at a measurable level in all the groups.

### IL-6

#### Controls vs IRI

During the 48 hour period, the production of IL-6 was increased in the IRI group compared to the control group, and was most marked at the 4 hour time-point, where an increase of 195% was noted (Table 1). Although this trend was observed over 48 hours, only at the 4 hour time-point was the production of IL-6 significantly higher (P = 0.028) in the IRI group compared to the control group.

**Table 1:** Production Level of IL-6 by SECs That Underwent IRI, IPC and IC at Their Respective Time Points

Time (Hours)	Control (Mean (SEM) pg/mL)	IRI		IPC		IC	
		Mean (SEM) pg/mL	P-value^ξ^	Mean (SEM) pg/mL	P-value^β^	Mean (SEM) pg/mL	P-value^χ^
0	4.14 (1.49)	7.03 (1.57)	NS	6.81 (1.61)	NS	5.82 (1.25)	NS
4	10.28 (1.24)	30.34 (7.39)	0.028	37.84 (9.39)	NS	29.28 (7.35)	NS
8	38.18 (9.90)	60.36 (11.74)	NS	66.79 (11.51)	NS	69.85 (23.62)	NS
12	45.49 (11.25)	73.14 (14.75)	NS	77.72 (14.71)	NS	75.03 (17.78)	NS
24	99.90 (35.02)	156.80 (43.62)	NS	139.68 (34.62)	NS	156.50 (42.78)	NS
36	180.62 (63.74)	253.83 (86.50)	NS	334.22 (145.38)	NS	309.92 (106.77)	NS
48	310.05 (126.36)	449.89 (135.11)	NS	572.22 (139.44)	NS	662.78 (149.64)	NS

^ξ^: P-value was derived from Mann Whitney U test between the IRI and control groups. ^β^: P-value was derived from Mann Whitney U test between the IPC and IRI groups. ^χ^: P-value was derived from Mann Whitney U test between the IC and IRI groups. NS: Not significant.

#### IRI vs IPC

Besides the 0 and 24 hour time-points, IPC exposure resulted in an increase in the production of IL-6 by SECs. The largest increase in IL-6 production recorded was 32% compared to IRI at the 36 hour time-point. Although there was a trend for IPC exposure to increase the production of IL-6 by SECs over 48 hours, none of these increases were statistically significant compared to IRI.

#### IRI vs IC

Following an initial decrease in the production of IL-6 up to 4 hours, there was an overall trend for IC to increase the profile of IL-6 up to 48 hours compared to IRI, with the most marked increase of 47% observed at the 48 hour time-point. However, IC did not significantly alter the profile of IL-6 compared to IRI during the 48 hour period.

### IL-8

#### Controls vs IRI

Overall, there was an increase in the production of IL-8 by SECs in the IRI group compared to the control group over the study period. Following IRI, the increase in IL-8 profile by SECs was most marked at the 4 hour time-point, where an increase of 131% was noted (Table 2). Nevertheless, this increase in IL-8 production by SECs following IRI was not statistically significant at all the time-points assessed over the 48 hour period compared to controls.

**Table2:** Production Level of IL-8 by SECs That Underwent IRI, IPC and IC at Their Respective Time Points

Time (Hours)	Control (Mean (SEM) pg/mL)	IRI		IPC		IC	
		Mean (SEM) pg/mL	P-value^ξ^	Mean (SEM) pg/mL	P-value^β^	Mean (SEM) pg/mL	P-value^χ^
0	108.78 (26.72)	118.58 (18.76)	NS	99.63 (18.34)	NS	100.43 (21.17)	NS
4	384.67 (95.76)	888.47 (332.47)	NS	885.73 (281.69)	NS	830.10 (262.55)	NS
8	993.77 (303.52)	1836.53 (750.05)	NS	1833.00 (812.49)	NS	1658.43 (552.36)	NS
12	1480.26 (630.49)	2318.59 (926.13)	NS	2318.41 (873.36)	NS	2244.22 (769.89)	NS
24	2339.97 (866.46)	3650.34 (1179.0)	NS	3727.84 (308.26)	NS	3811.90 (1282.7)	NS
36	495.56 (177.68)	528.10 (161.22)	NS	613.32 (178.73)	NS	695.27 (172.94)	NS
48	686.15 (336.40)	763.70 (366.15)	NS	572.22 (139.44)	NS	842.01 (238.24)	NS

^ξ^: P-value was derived from Mann Whitney U test between the IRI and control groups. ^β^: P-value was derived from Mann Whitney U test between the IPC and IRI groups. ^χ^: P-value was derived from Mann Whitney U test between the IC and IRI groups. NS: Not significant.

#### IRI vs IPC

During the first 12 hours following IPC, IL-8 production levels by SECs were decreased compared to IRI. After 24 hours, there was an increase in IL-8 production recorded, up to 16% compared to IRI at the 36 hour time-point. Nevertheless, IPC exposure did not significantly influence the production of IL-8 by SECs over the 48 hour period compared to IRI.

#### IRI vs IC

In comparison to the IRI group, there was a trend for the IC group to decreases the production of IL-8 by SECs in the first 12 hours and increase the production thereafter. However, IC did not significantly influence the profile of IL-8 during the 48 hour study period.

### G-CSF

#### Controls vs IRI

The exposure of IRI on SECs led to an increase in G-CSF production throughout the study period compared to controls. At the 4 hour time-point following IRI, the increase in G-CSF production was most marked, at 124% compared to controls. Although there was a trend for the IRI group to increase the production levels of G-CSF over 48 hours, this increase was not statistically significant compared to the control group.

#### IRI vs IPC

During the initial 24 hours, G-CSF production levels by SECs were reduced in the IPC group compared to the IRI group. The reduction in G-CSF levels during this period ranged from 10% to 15%. However, after 24 hours, there was an increase in G-CSF production by 19% and 40% at the 36 and 48 hour time-points, respectively compared to IRI (Table 3). However, IPC exposure did not significantly alter the production of G-CSF by SECs over the study period compared to IRI.

**Table 3:** Production Level of G-CSF by SECs That Underwent IRI, IPC and IC at Their Respective Time Points

Time (Hours)	Control (Mean (SEM) pg/mL)	IRI		IPC		IC	
		Mean (SEM) pg/mL	P-value^ξ^	Mean (SEM) pg/mL	P-value^β^	Mean (SEM) pg/mL	P-value^χ^
0	3.79 (1.25)	7.53 (1.55)	NS	6.73 (1.20)	NS	4.44 (0.60)	NS
4	11.44 (5.91)	25.67 (5.53)	NS	22.47 (6.65)	NS	19.90 (5.23)	NS
8	40.61 (17.39)	74.36 (22.74)	NS	67.11 (23.69)	NS	63.75 (20.32)	NS
12	70.74 (31.93)	108.61 (31.76)	NS	92.37 (26.42)	NS	101.80 (28.18)	NS
24	159.52 (65.94)	234.59 (73.61)	NS	208.74 (59.72)	NS	243.60 (63.22)	NS
36	214.30 (88.84)	330.58 (99.61)	NS	392.03 (133.34)	NS	432.04 (152.23)	NS
48	306.84 (116.61)	467.89 (128.72)	NS	656.95 (253.13)	NS	719.84 (268.52)	NS

^ξ^: P-value was derived from Mann Whitney U test between the IRI and control groups. ^β^: P-value was derived from Mann Whitney U test between the IPC and IRI groups. ^χ^: P-value was derived from Mann Whitney U test between the IC and IRI groups. NS: Not significant.

#### IRI vs IC

With respect to G-CSF production over 48 hours, IC had a negative effect on its production during the first 12 hours in comparison to IRI. Thereafter, IC gradually increased the production of G-CSF between the 24 and 48 hour time-points, from 4% to 54% compared to the IRI group. Nonetheless, IC did not significantly influence the concentration levels of G-CSF compared to the IRI group.

### HGF

#### Controls vs IRI

There was an overall increase in the production of HGF levels in the IRI group compared to the control group (Table 4). Although these increases in HGF levels were observed over the study period, there were no significant differences in the production of HGF between the IRI and control groups.

**Table 4:** Production Level of HGF by SECs That Underwent IRI, IPC and IC at Their Respective Time Points

Time (Hours)	Control (Mean (SEM) pg/mL)	IRI		IPC		IC	
		Mean (SEM) pg/mL	P-value^ξ^	Mean (SEM) pg/mL	P-value^β^	Mean (SEM) pg/mL	P-value^χ^
0	2.57 (0.76)	4.40 (0.56)	NS	4.74 (1.83)	NS	6.58 (2.36)	NS
4	6.36 (2.83)	13.40 (2.44)	NS	19.34 (7.34)	NS	21.88 (5.53)	NS
8	16.86 (6.58)	25.36 (5.06)	NS	43.28 (16.26)	NS	34.04 (11.41)	NS
12	15.45 (3.19)	35.56 (8.15)	NS	49.16 (16.99)	NS	47.00 (12.76)	NS
24	28.65 (8.87)	48.28 (11.38)	NS	60.82 (23.02)	NS	83.95 (25.26)	NS
36	25.73 (8.20)	62.64 (12.96)	NS	119.69 (26.44)	NS	132.00 (11.89)	NS
48	110.16 (50.80)	171.78 (50.19)	NS	182.70 (39.07)	NS	208.52 (27.50)	NS

^ξ^: P-value was derived from Mann Whitney U test between the IRI and control groups. ^β^: P-value was derived from Mann Whitney U test between the IPC and IRI groups. ^χ^: P-value was derived from Mann Whitney U test between the IC and IRI groups. NS: Not significant.

#### IRI vs IPC

During the study period, there was a trend for IPC prior to IRI to increase the production of HGF by SECs, in particular at the 8 and 36 hour time-points, where an increase of 71% and 91% were respectively noted. Despite this trend, there was no significant difference in HGF production between the IPC and IRI groups.

#### IRI vs IC

Following the application of IC, the production of HGF by SECs was increased throughout the 48 hour period compared to IRI, in particular at the 12 and 36 hour time-points, where an increase of 74% and 111%, was respectively observed. However, there was no significant difference in HGF production between the IC and IRI groups.

## Discussion

Following liver surgery, successful patient outcome often depends on liver regeneration, particularly in patients with cirrhotic and steatotic livers. Regeneration of the liver following IRI and major liver surgery is a complex process that involves the integration of a network of cytokines and growth factors. The up-regulation of cytokines and growth factors involved in liver regeneration by surgical methods is an interesting and feasible option. The beneficial effects of IPC [11] and IC [12] on the liver following IRI has been described previously, and currently their role in liver regeneration are being evaluated. In this study, isolated human liver SECs in culture produced IL-6, IL-8, G-CSF and HGF, and their production was not significantly influenced by IPC and IC.

### IL-6

IL-6 plays a crucial role in the initiation of the acute phase response in the liver following hepatic resection, and the absence of this response has been shown to impair liver regeneration [13]. IL-6 is produced by SECs and is thought to act directly on hepatocytes by binding to the IL-6 receptor complex and induces the translocation of STAT-3. This leads to a cascade of events that allows progression of the cell cycle, and subsequent cellular mitosis [14].

The effect of IPC on the production of IL-6 following partial hepatectomy has produced conflicting results. Some authors have observed an increase in IL-6 profile following IPC. Using a partial hepatectomy model in rats, Bedirli et al observed a trend for IPC to increase serum IL-6 levels during the late (24 and 48 hours) phases of reperfusion [15]. Other authors have also shown an increase in hepatic IL-6 mRNA and STAT-3 levels at 24 hours of reperfusion following total hepatic ischaemia [16].

Previous reports have also demonstrated that IPC inhibits IL-6 production. Following partial hepatectomy in rats, an early increase in the levels of IL-6 seen in controls, was absent in the IPC group [15]. Tsuyama et al also reported a significant decrease in IL-6 production at the 2 and 5 hour period of reperfusion following IPC (15 minutes of ischaemia and 20 minutes of reperfusion) in mice subjected to hepatic ischaemia [17].

In the current study, IPC did not significantly modify the levels of IL-6 profile in SECs in-vitro. Similarly, Franco-Gou and co-investigators reported that IPC did not influence the levels of IL-6 and TNF-α in reduced size liver for transplantation in rats [18]. Chouker et al observed no significant differences in plasma IL-6 concentrations in patients undergoing liver resection without the Pringle maneuver; with Pringle maneuver; and IPC prior to Pringle maneuver [19]. This may suggest that surgical trauma alone is the major factor accounting for the release of IL-6 to which IRI does not add any significant difference. Furthermore, IPC application did not significantly influence the profile of IL-6 after liver resection and IRI, suggesting that IPC does not influence the systemic profile of IL-6 following hepatectomy. The differences in results between these studies may be due to the disparity in IPC times used and experimental design employed (in-vivo versus in-vitro). Nevertheless, the role of IPC in influencing the profile of IL-6 should not be discounted.

With respect to IC, Kimura et al noted undetectable levels of IL-6 at 2 hours in the IC (15 minutes ischaemia and 15 minutes reperfusion) group in a hepatic ischaemia rat model of 60 minutes [12]. Using a similar model with hepatic ischaemia of 120 minutes, there was initially a significantly higher systemic IL-6 profile at the 0 hour time-point, followed by a significantly lower IL-6 level at the 3 and 5 hour time-points in the IC (30 minutes ischaemia and 5 minutes reperfusion) group compared to the continuous clamping group [20]. However, the present study did not observe any significant differences in IL-6 profile following IC.

### IL-8

Besides being involved in neutrophil-mediated injury following hepatic IRI, IL-8 also functions as a survival factor for liver cells [21]. The underlying mechanism responsible for IL-8 mediated survival function in human hepatocytes is thought to be due to TNF-α signalling via the NF-κB and the phosphatidylinositol 3-kinase / Akt pathway [21]. This signalling pathway is also crucial in the liver regeneration process. Colletti et al investigated the role of IL-8 on rat hepatocytes proliferation in-vitro and demonstrated a significant increase in proliferation following exposure to IL-8 [22].

There are few published studies assessing the effect of IPC on the production of IL-8 following liver surgery and IRI, but none on liver regeneration. In patients undergoing liver resection, Chouker and co-workers reported no statistically significant difference in systemic IL-8 production in patients pre-treated by IPC compared to patients who were not subjected to IPC [19]. There is also currently no available data on the effect of IC on IL-8 following liver surgery, IRI and regeneration. In the present study, IPC and IC did not significantly influence the IL-8 production in SECs in-vitro.

### G-CSF

G-CSF is a haematopoietic growth factor that stimulates proliferation and differentiation of neutrophil colony-forming cells, and previous reports have observed that G-CSF promotes liver regeneration in rodents. Inderbitzin et al demonstrated that G-CSF supported liver regeneration in a small for size liver remnant mouse model [23]. More recently, Piscaglia and co-workers demonstrated that G-CSF facilitated hepatic regeneration in rats subjected to partial hepatectomy by increasing the migration of bone marrow-derived progenitors to the liver, as well as enhancing the endogenous liver stem cell reaction [24]. Hence, G-CSF has both a local and systemic effect on the liver regeneration process.

At present, there are no published studies assessing the effect of IPC and IC on the production of G-CSF following hepatectomy or liver transplantation. Nonetheless, the current study did not show any significant difference in the profile of G-CSF following both IPC and IC exposure compared to IRI in SECs in-vitro.

### HGF

HGF is known to be a potent mitogen for mature parenchymal rat hepatocytes in primary culture [25], and seems to be a hepatotrophic factor that acts as a trigger for liver regeneration following liver resection and liver injury [18, 26].

Franco-Gou and investigators observed a significant increase in HGF levels after 24 hours following IPC prior to reduced-size orthotopic liver transplantation in rats compared to controls [27]. IPC has also been shown to increase both liver and plasma HGF levels in reduced size liver transplantation rat models, and this was associated with an increase in hepatocyte proliferation [18]. These results suggest that IPC influences HGF profile after 24 hours and is likely to enhance the HGF-mediated signalling pathways of the regeneration process at this time-point. In contrast, the present study did not demonstrate a significant effect of IPC on the production of HGF in SECs in-vitro.

There are currently no published studies assessing the outcome of HGF profile following IC exposure during liver resection and transplantation. In this study, IC did not significantly alter the production of HGF in isolated SECs in-vitro.

### IPC versus IC

To date, there have been few in-vivo and human studies comparing the efficacy of both these strategies. In addition, none of these studies focused on the effect of these surgical techniques on liver regeneration.

Using a 65% hepatectomy pig model, Kadono et al observed significantly lower levels of transaminases, improved hepatic micro-circulation and reduced hepatocyte necrosis on histopathology analysis in pigs treated with IPC compared to controls [28]. In comparison to IC, IPC significantly reduced the plasma levels of TNF-α and endothelin-1 (potent vasoconstrictor) at 30 and 120 minutes following reperfusion, leading the authors to conclude that IPC was more effective in decreasing the inflammatory response following hepatectomy compared to IC [28]. Other groups have suggested that both IC and IPC equally effective in increasing the tolerance of fatty livers to hepatic IRI. In a steatotic rat liver model of 75 minutes of hepatic ischaemia and 3 hours of reperfusion, Saidi and co-workers reported significantly lower hepatocellular injury and serum IL-6 levels with IPC and IC compared to controls [29]. A recent meta-analysis of the effect of portal triad clamping on outcome following liver resection revealed no significant difference between IPC and IC in post-operative outcome [30]. However, this meta-analysis consisted of only eight randomised control trials, considerable differences between study designs, no subgroup analysis of patients with cirrhosis and no long-term follow-up data.

### Conclusion

In the present study, both IPC and IC failed to significantly influence the production of IL-6, IL-8, G-CSF and HGF using an in-vitro human SECs model. Nevertheless, the potential clinical benefit of IPC and IC in major liver surgery should not be discounted. The role of surgical techniques in influencing IRI and liver regeneration requires further evaluation and whether IPC is superior to IC or vise versa remains unknown. The assessment of IPC and IC on liver regeneration in human studies is clearly the next step.
